# Faith Moves Mountains—Mountains Move Faith: Two Opposite Epidemiological Forces in Research on Religion and Health

**DOI:** 10.1007/s10943-016-0300-1

**Published:** 2016-08-19

**Authors:** N. C. Hvidt, D. Hvidtjørn, K. Christensen, J. B. Nielsen, J. Søndergaard

**Affiliations:** 1 Research Unit of General Practice, Institute of Public Health, University of Southern Denmark, J. B. Winsløwsvej 9A, 5000 Odense, Denmark; 2 Institute of Clinical Research, University of Southern Denmark, Odense, Denmark; 3 Epidemiology, Biostatistics and Biodemography, Department of Public Health, University of Southern Denmark, Odense, Denmark; 4 Department of Clinical Biochemistry and Pharmacology and Department of Clinical Genetics, Odense University Hospital, Odense, Denmark; 5 The Danish Twin Registry, University of Southern Denmark, Odense, Denmark

**Keywords:** Spirituality and health, Religious coping, Religious seeking, Religious struggle, Meaning-making

## Abstract

Research suggests opposite epidemiological forces in religion and health: (1). Faith seems to move mountains in the sense that religion is associated with positive health outcomes. (2). Mountains of bad health seem to move faith. We reflected on these forces in a population of 3000 young Danish twins in which all religiosity measures were associated with severe disease. We believe the reason for this novel finding is that the sample presents as a particularly secular population-based study and that the second epidemiological force has gained the upper hand in this sample. We suggest that all cross-sectional research on religion and health should be interpreted in light of such opposite epidemiological forces potentially diluting each other.

## Introduction

*Does Faith Move Mountains?* Results from a body of research on religion, spirituality and health that has grown “explosively” (Hall et al. 2004) over the past two decades suggest an affirmative answer. The majority of basic religiosity measures are associated with positive mental and bodily health outcomes (Koenig et al. 2012; Pargament 2013) and longevity (H. G. Koenig 2006). Former reviews have shown that 72 % of studies have shown positive relationships between religion and mental health, 16 % have shown negative relations, and 12 % have shown no relations (Bonelli and Koenig 2013; Larson et al. 1986, 1992). Researchers explain the findings by healthy lifestyle, positive psychological resources, perceived purpose, personal meditation and prayer, day of rest and community network, (Harold G. Koenig et al. 2012; Strecher 2016) but also more sophisticated psychological causal pathway models (Aldwin et al. 2014; Levin et al. 2011). Although the clinical implications of this research remain controversial (Sloan 2006) and although conceptualizing and measuring “religion” and “spirituality” remain a challenge (Hall et al. 2004), the basic results indicating a positive association between religion and health remain undisputed.

Conversely, research suggests that *Mountains Move Faith.* Nothing seems to propel, activate and intensify religious seeking as much as mountains of crisis and disease (Ferraro and Kelley-Moore 2000). Some studies employing religious coping measures (such as personal prayer or finding religion to be of high importance) are associated with higher incidence of depression: People who are depressed or faced with a problem generally pray more and find faith in God to be more important (Nicholson et al. 2010).

**Fig. 1:**
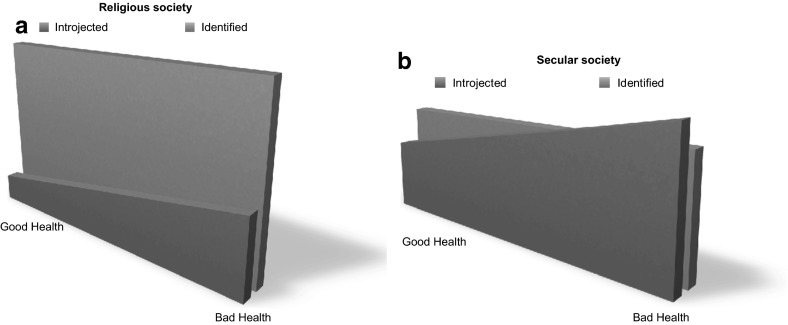
Two figures illustrate the relative difference of crisis and restful religiosity in religious and secular societies. In both societies, crisis religiosity will increase with increased illness (**a**), but in secular societies, crisis religiosity will eventually overcome restful religiosity (**b**)

We conceptualized these two tendencies in cross-sectional research on religion and health as opposite forces potentially canceling or diluting each other and that the extent of each force is skewed as only one combined result of both forces is visible: In every given population investigated for its religiousness, there will be some that are mainly religious because they rest intrinsically in their faith, whereas the religiosity of others may mainly be propelled by disease and crisis. Building on existing research, we further conceptualized the two tendencies as two inseparable aspects, even forms, of religion and religious internalization that may be present in most types of believers; some believers may be more marked by the first aspect/form, others more by the second. The first is more pronounced in religious societies such as the USA, and the second more present in secular societies, such as those of Northern Europe. The first tendency/form has been described as “internalization through identification” where belief is adopted as personal volition and value, adopted by longstanding reliance on religion and marked by restfulness (hereafter “restful religiosity”), whereas the opposite “internalization through introjection” is provoked by group pressure or by stress and comes across as increased reliance on religion during crisis (hereafter “crisis religiosity”) (Ryan et al. 1993) (Fig. 1). We thus assumed that the more secular a given population would be with low degrees of religious belief, practice and importance, the more likely it would be that the second force would gain the upper hand over the first: Eventually, the incentives for maintaining the first form of religiosity that rests in itself and is able to move mountains of health might cede leaving the “cross-sectional scene” to be dominated by the second form where faith is activated by mountains of disease and crisis; given a high enough rate of secularity, *crisis religiosity* might eventually win over *restful religiosity* and become the predominant epidemiological force in this cross-sectional “force field” (see Fig. 1). This is supported from a recent study from China: Researchers examining a largely secular population in western Mainland China found that in secular countries like China, there must be considerable stress and trauma for people to turn to religion as a coping behavior since it is not supported by the general population. In such cases, religiosity may actually represent a “marker” of distress or illness, thus concealing some of the health benefits derived from it (Wang et al. 2016).

## Method

### Setting and study population

We now present data that for the first time lends substantial credibility to this assumption from a population-based study that presents as a particularly secular population. The population is a sample of 3000 young Danish twins taking part in a large interdisciplinary study on the genetic influence on attitudes and values in general, including ethical, political and religious questions that has been detailed earlier (Hvidtjørn et al. 2014, 2013; Klemmensen et al. 2012), and now we report the findings on the association between religiosity and health in the sample.

In October 2009, we sent an invitation to 6707 monozygotic and dizygotic twins born 1970–1989 to participate in an online survey. Participants could request a paper version, if they preferred. Nine questions had previously been used in the 2008 European Values Study (EVS) (Gundelach 2008), a much-used and quoted survey on views and values in general, including political and ethical convictions, experiences of life crises, religious beliefs and existential values. Five new items on religiousness and coping with crisis were developed for the present survey. The questionnaire was preceded by questions on age, gender, health status and educational attainment.

### Covariates in Analysis

Variables on religiousness: We organized religiousness on existing research (La Cour and Hvidt 2010) suggesting that religiosity has three important dimensions: Cognition—related to beliefs and convictions, Practice—related to the practices affiliated with these beliefs, and Importance—related to the experienced significance these beliefs have in the life of a person. *Beliefs in God (yes/no)* and *Life after death (yes/no)* were conceived as Cognition, *Frequency of personal prayer* (once a week or more/less than once a week) and *Religious attendance (once a month or more/less than once a month)* as Practice, and *Importance of God* and *Finding comfort and strength in religion* as Importance (the latter two dichotomized into yes or no, from a ten points Likert scale divided at the five point).

Variables on health and crisis included: Serious disease diagnosed by a medical doctor (cancer and epilepsy), regular use of medication (excluding vitamins and contraception), chronic disease (self-reported), self-rated health dichotomized in good (excellent, very fair and fair) versus bad (not so fair and bad), life-threatening disease (self-reported) and having experienced what the respondents themselves considered a life crisis.

### Statistical Analysis

Associations between religiousness and experiences of crisis and disease were expressed in relative risks (RR) with 95 % confidence intervals estimated by binominal regression, crude and adjusting for age (continuous), gender and educational attainment in four categories (none (reference), <3, 3–4, more than four years). The CIs were adjusted for dependence within twin pairs. STATA, version 11.2, was used in the processing of data.

## Results

In total, 3686 twins completed the survey resulting in an overall response rate of 55 %. The online version of the questionnaire was answered by 3652 with only 34 twins using the paper version. Exactly 3000 answered the questions on religion and crisis resulting in a response rate of 45 % for this section. The vast majority (82.6 %) were members of the Danish National Lutheran Church, corresponding exactly to the national level of 2009 (Ministry of Ecclesiastical Affairs 2015). 60 % of the respondents were women, and nearly twice as many men as women were not religiously affiliated. In this respect, the sample reflects Danish culture.

Although membership of the Evangelical Lutheran Church in Denmark equaled average, all other measures portrayed the sample as particularly secular. When comparing the sample with the US population, a population Denmark is often considered similar to socioeconomically, only 6 % of the twins reported attending church once a month or more vs 73 % of US Americans, and when 98 % of the American sample state that they believe in God, this is only true for 41 % of the twins (Table 1).

**Table 1:** Percentages of people in Denmark, Great Britain and the USA answering Yes to questions on religiousness in the dimensions; Cognition, Practice and Importance, doing and being

Answering yes, percentages (Excluding “don’t know”)	Dimension of religiosity	DK Twins^a^	DK^b^	Great Britain	USA^c^
Do you believe in God?	Cognition	41 %	64 %	68 %^b^	89 %
Do you believe in life after death?	Cognition	42 %	36 %	55 %^b^	72 %^*^
Do you pray once a week or more?	Practice	11 %	17 %	29 %^b^	66 %
Do you attend Church once a month or more?	Practice	6 %	10 %	23 %^c^	44 %
Do you find comfort in religion?	Importance	26 %	35 %	42 %^b^	69 %^**^
Is God important in your life? Answering on a Likert scale from 0 to 10, dichotomized at 5	Importance	19 %	27 %	50 %^c^	79 %

^a^From the present study

^b^From The European Values Study 2008

^c^From The World Values Survey 2010–2014

* “Do you believe in hell?”

** “Do you find religion important?”

Nevertheless, the sample proved to be even less religious than the average Danish population, as measured in the EVS. Only half as many twins as Danes in general attended church once a month or more, and while 17 % of the Danish population pray to God at least weekly, this was only the case for 11 % of the twins (Table 1).

Contrary to the findings of almost all cross-sectional studies on religion and health (Harold G. Koenig et al. 2012), the present cross-sectional study found religiousness to be associated with experiences of crisis and poor health. Regardless which of the three religiosity dimension we gauged, we found associations with bad health, though not significant in all health measures: In the Cognition dimension, only life-threatening disease was significantly associated with belief in God, whereas in the Practice dimension, nearly all measures of poor health and crisis were significantly associated with praying to God at least weekly. Likewise, the religiosity measure most often and most powerfully found to be associated with reduced risk of disease and with longevity; namely, church attendance (Hill and Pargament 2008; Harold G. Koenig et al. 2012, p. 488; Powell et al. 2003), was, in our sample, associated with increased risk of chronic or life-threatening disease. In the Importance dimension, nearly all measures of health were strongly associated with both importance of God and finding comfort and strength in religion (Table 3). Of the 3000, who replied to the survey section on religion, crisis and coping, 1838 (61 %) reported to have had a crisis experience that they experienced as “heavy to bear.” Over half of these replied affirmatively to the question “Has the crisis made you think more about religious questions?” (Table 2).

**Table 2:** Numbers and percentages of how much a crisis changed thoughts about religious questions among the twins who did experience a crisis

Has the crisis made you think more about religious questions?	
Not at all	870 (47 %)
A little	397 (22 %)
Some	402 (22 %)
Very much	169 (9 %)

## Discussion

We have reported a cross-sectional sample that over a range of religiosity measures comes across as a particularly secular population. There may be at least three reasons why the population is so secular.

First, Denmark is known as one of the least religious countries in the world and as a “Society without God” (Zuckerman 2008). Although Denmark has the highest membership rate of any national church with 78 % of Danes being voluntary tax-paying members of the Danish National Lutheran Church as of today (82.6 % in 2009), (Ministry of Ecclesiastical Affairs 2015) only 10 % of Danes attend church on a monthly basis (vs 44 % in the USA), amounting to one of the lowest rates of attendance worldwide (Table 1). Another significant indicator of secularity is belief in an “absolute truth”: 95 % of Pakistanis, 70 % of US Americans, 40 % of British but only 10 % of Danes believe in an absolute truth (Raun Iversen et al. 2008).

Second, the present population is rather young (between 20 and 40 years of age 1 November 2009), and young people tend to be less religious. The medieval Italian poet Ludovico Ariosto is quoted for saying: “When the devil grows old he turns hermit.” This saying reflects a popular notion that people turn to faith the more they age and approach death, and hence that young people are generally less religious than older. Methodically diverse research confirms the scientific foundation of this notion (Levin et al. 2011).

Third, twins may be less religious than singletons. Although studies have identified twins as representative for the population at large (Johnson and Zhang 2002) some studies suggest that having a twin may reduce the risk of suicide, possibly due to the secure twin-fellowship (Tomassini et al. 2003), which might also lower the experienced need for Divine fellowship.

Given that our sample proved to be even more secular than the average Danish (secular) society, we were curious to see whether we would find here a population in which our assumption would be sustained that religious people in rarely secular societies would be more ill than their less religious peers as the primary driving force for religion in such society is crisis?

So far the only large-scale study on religiosity and health status in Denmark has but confirmed the US American results on religion and health. In this sample of 12,000 Baptists and Adventists in Denmark, both groups had a significantly lower risk of diseases such as cancer (40 % overall reduction) and in particular lifestyle-related diseases such as lung cancer (72 % reduction) (Thygesen et al. 2012). In another cohort of 734 men and women born 1914 in Glostrup, Denmark, women who attended church “once in a while” lived on average 2.6 year longer than women who did not. The effect was reduced but remained significant when correcting for known confounders such as smoking, alcohol and BMI (La Cour et al. 2006).

However, these two samples are not representative of the general Danish Population. Baptists and Adventists are a minority in Denmark known exactly for their unusual religious involvement, as are most religious minorities (Finke and Stark 1998). The Glostrup cohort consists of elder people known to be generally more religious than younger/average populations and may have grown up in a time when religion was to a larger degree part of society than today.

Conversely, all primary religiosity measures in our study are associated with crisis and bad health: The religious in the sample are more ill than their nonreligious peers. In fact, all most-used religiosity measures across the three religiosity dimensions of Cognition, Practice and Importance are associated with experienced crisis, chronic illness and life-threatening disease. Although our study does not contain particular measures for restful vs crisis religiosity, our careful interpretation of this data is that we may have with this population a situation where crisis religiosity has gained the upper hand over restful religiosity that is normally associated with good health outcomes. This interpretation is further strengthened by the aforementioned finding that over half of respondents thought more about religious questions due to experienced crisis and that the religiosity measure most powerfully associated with illness was “Finding comfort in God” (Tables 2 and 3).

**Table 3:** Associations between measures of health and religious items in relative risks, crude and adjusted for gender, educational level and age, clustered

	Cognition			
	Believe in God 983 (41 %)		Believe in life after death 955 (42 %)	
	Crude	Adjusted	Crude	Adjusted
Experienced a crisis 1902 (67 %)	1.12 (1.00–1.25)	1.03 (0.92–1.15)	1.18 (1.05–1.34)	1.06 (0.94–1.20)
Medicine use 734 (24 %)	1.10 (0.99–1.23)	1.02 (0.92–1.14)	1.10 (0.98–1.23)	1.04 (0.94–1.16)
Serious disease 209 (7 %)	1.02 (0.85–1.22)	0.95 (0.80–1.43)	1.20 (1.01–1.41)	1.07 (0.90–1.27)
Chronic disease 94 (3 %)	1.24 (0.97–1.58)	1.15 (0.90–1.48)	1.28 (1.02–1.60)	1.16 (0.91–1.48)
Bad self–rated health 111 (4 %)	1.18 (0.94–1.47)	1.15 (0.93–1.43)	1.37 (1.13–1.67)	1.34 (0.90–1.44)
Life–threatening disease 92 (3 %)	1.35 (1.11–1.66)	1.27 (1.04–1.54)	1.11 (0.86–1.43)	0.92 (0.69–1.23)

	Practice			
	Pray to God weekly 319 (11 %)		Go to church monthly 169 (6 %)	
	Crude	Adjusted	Crude	Adjusted
Experienced a crisis 1902 (67 %)	1.65 (1.26–2.16)	1.67 (1.25–2.23)	1.32 (0.91–1.91)	1.35 (0.92–1.20)
Medicine use 734 (24 %)	1.33 (1.11–1.59)	1.27 (1.06–1.53)	1.07 (0.74–1.51)	1.08 (0.75–1.56)
Serious disease 209 (7 %)	1.20 (0.79–1.83)	1.06 (0.69–1.64)	1.51 (0.89–2.55)	1.30 (0.74–2.29)
Chronic disease 94 (3 %)	1.98 (1.15–3.42)	1.98 (1.15–3.42)	2.57 (1.47–4.65)	2.68 (1.47–4.88)
Bad self-rated health 111 (4 %)	1.75 (1.06–2.90)	1.83 (1.05–3.02)	1.48 (0.73–2.99)	1.59 (0.78–3.22)
Life-threatening disease 92 (3 %)	1.76 (1.04–2.98)	1.86 (1.10–3.15)	1.98 (1.09–3.62)	2.12 (1.17–3.88)

	Importance			
	Find comfort 681 (26 %)		Importance of God 570 (19 %)	
	Crude	Adjusted	Crude	Adjusted
Experienced a crisis 1902 (67 %)	1.55 (1.32–1.83)	1.46 (1.23–1.73)	1.51 (1.26–1.82)	1.45 (1.19–1.77)
Medicine use 734 (24 %)	1.20 (1.04–1.39)	1.12 (0.96–1.0)	1.07 (0.90–1.27)	1.00 (0.84–1.20)
Serious disease 209 (7 %)	1.15 (0.91–1.45)	1.13 (0.98–1.49)	1.07 (0.80–1.44)	1.01 (0.75–1.36)
Chronic disease 94 (3 %)	1.60 (1.22–2.12)	1.57 (1.19–2.08)	1.52 (1.08–2.16)	1.47 (1.03–2.09)
Bad self-rated health 111 (4 %)	1.62 (1.25–2.10)	1.63 (1.26–2.11)	1.36 (0.98–1.89)	1.30 (0.92–1.83)
Life-threatening disease 92 (3 %)	1.41 (1.05–1.90)	1.44 (1.06–1.95)	1.74 (1.29–2.36)	1.60 (1.16–2.20)

Numbers and percentages of twins answering “Yes” in the health and religious measures

Strengths of our study were the large sample size and the diverse and multiple measures of religiosity. The main limitation was a somewhat meager response rate of 55 % overall and 45 % for the section on religiosity and crisis. This could skew our study in two opposite ways: First, those with religious interests could be more prone to answer the study, but this is unlikely given that our sample overall is only half as religious as are typical Danes that may well identify themselves “believer” but hardly “religious” (Rosen 2009); conversely one could expect that those who are ill might be underrepresented as their disease or crisis would deter them from responding, but very few aged 25–35 are seriously ill. This could actually strengthen our conclusions as the associations between religiosity and bad health might then actually be underreported. Further, some information on health status (health status, chronic disease and life-threatening disease) was self-reported which could be biased, although the trends were the same for serious disease diagnosed by a medical doctor (cancer and epilepsy). Finally, as mentioned twins are known to have a lower risk of suicide, probably because they support each other and hence would be less likely to seek divine support. This could potentially dilute our study’s finding of a dominance of crisis religiosity as twins may be in lesser need for divine support during crisis than singletons as they rely on each other for support, which in turn would strengthen our case: Was it not for the mutual support of twins, the prevalence of crisis religiosity would have been expected to be even larger in our sample. Our study would have profited from a longitudinal design, as we cannot draw definitive conclusions from cross-sectional design. So far, however, such design has not been possible with this twin sample.

### The Implications of this Study are at Least Fourfold

First, there may be an interaction of the two “forces of an epidemiology of religion” in most cross-sectional studies. The “negative, crisis force” that may for the first time have been documented cross-sectionally would have been probably even stronger were it not for those individuals in our sample that were religious in the restful way. Consequently, the evidence for a positive association between religiosity and health published so far from more religious populations would actually be larger than what these studies have reported due to such diluting or out canceling effect that has only been accounted for sporadically in research.

Second, the more secular a country grows, the larger the proportion of actively religious people adhering to crisis religiosity may become.

Third, the crisis religiosity identified in our study, which has not been internalized in a restful way prior to the crisis, may not be very secure and fruitful. International studies suggest that people who prior to a given crisis are insecure about and have not internalized their religiosity in a restful way tend to develop negative religious coping patterns that actually increase the risk of disease-related depression(Fitchett et al. 2004) as their coping pattern constitutes an unprepared and untrained coping resource. This would suggest that health care attention to the spiritual needs and pains of sufferers (World Health Organization 2002) would actually be more warranted in particularly secular nations as fewer patients would enter the field of crisis with the robust resource of a restful, well-prepared religiosity that would stand its ground in crisis and disease.

Fourth, more studies should be developed including questions better aimed at identifying the two types of religiosity presented in this study in order to evaluate (a) How crisis religiosity develops over time, (b) Whether it transforms and persists in the more restful way, even after the disease crisis. Likewise, prospective studies should be performed to evaluate how each of the two types of religiosity may be associated with good or bad health.

The implication of the present observation is potentially controversial in secular societies. Could societal support to or collaboration with faith-based health care or initiatives–found to be so vital in third world countries (“Lancet Series on Faith-Based Health Care,” July 7 2015)—constitute a cost-effective supplement to some of the traditional care offered to people struck by crisis or disease, even in secular society?
